# Proteotoxic crisis, the ubiquitin-proteasome system, and cancer therapy

**DOI:** 10.1186/s12915-014-0094-0

**Published:** 2014-11-11

**Authors:** Raymond J Deshaies

**Affiliations:** Division of Biology and Biological Engineering and Howard Hughes Medical Institute, California Institute of Technology, Box 114–96, Pasadena, CA 91107 USA

## Abstract

Genomic alterations may make cancer cells more dependent than normal cells on mechanisms of proteostasis, including protein folding and degradation. This proposition is the basis for the clinical use of proteasome inhibitors to treat multiple myeloma and mantle cell lymphoma. However, proteasome inhibitors have not proved effective in treating other cancers, and this has called into question the general applicability of this approach. Here, I consider possible explanations for this apparently limited applicability, and discuss whether inhibiting other broadly acting components of the ubiquitin-proteasome system - including ubiquitin-activating enzyme and the AAA-ATPase p97/VCP - might be more generally effective in cancer therapy.

## The ubiquitin-proteasome system and the ‘proteotoxic crisis’ approach to cancer therapy

The ubiquitin-proteasome system (UPS) is the major mechanism by which proteins are degraded in the cytoplasm and nucleus of eukaryotic cells and as such is a key player in maintaining protein homeostasis [1]. Proteins destined to be degraded by the UPS are tagged for destruction by conjugation to the small protein ubiquitin through the action of ubiquitin-conjugating (E2) and ubiquitin ligase (E3) enzymes, which can result in the assembly of ubiquitin chains on one or more lysine residues within the substrate. Proteins modified with an ubiquitin chain bind to ubiquitin receptors that link them to the 26S proteasome. The 26S proteasome is a large proteolytic complex that degrades ubiquitin-modified proteins and recycles the ubiquitin for future use.

**Figure 1:**
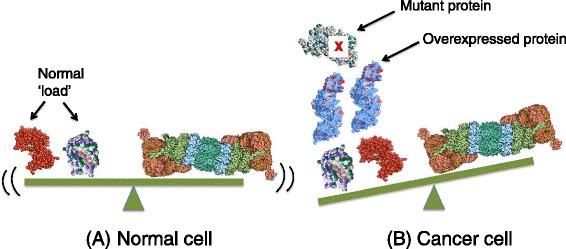
**Proteotoxic crisis in cancer cells. (A)** In normal cells, the natural load of degradation substrates on the left is in balance with the capacity of the cellular ubiquitin-proteasome system (UPS), signified by the proteasome on the right. **(B)** In cancer cells, the load is increased due to expression of mutant proteins and/or expression of excess proteins due to aneuploidy. This results in an imbalance where the degradation load exceeds the capacity of the UPS.

Several lines of evidence suggest that cancer cells have a heightened dependence on mechanisms of protein homeostasis (proteostasis) [2], including the UPS (Figure 1). Genome sequencing has revealed that cancer genomes are typically littered with dozens to hundreds of point mutations in protein coding sequences [3]. Many of these mutated proteins are likely to present significant folding challenges, with increased degradation of the mutant protein via the UPS being one possible outcome. In addition, cancer cell genomes often contain large duplications, deletions, inversions, and translocations as well as altered copy numbers of entire chromosomes (aneuploidy). It has been estimated that over 90% of human solid tumors contain cells with more than two copies of one or more chromosomes [4]. These excess chromosomes continue to be expressed, and therefore protein synthesis in aneuploid cancer cells is often imbalanced, with proteins encoded by extra chromosomes being produced in excess over proteins encoded by chromosomes that are present in two copies [5,6]. This is particularly a problem for proteins that assemble to form stoichiometric complexes like the ribosome. In such cases, the excess proteins almost certainly cannot attain stable conformations, and hence are degraded by the UPS [7,8]. In theory, this creates in cancer cells a heightened dependence on protein quality-control (PQC) mechanisms, including protein chaperones, the UPS, and autophagy [9-12]. In agreement with this, approximately one-third of single chromosomal aneuploidies in yeast cells render them hypersensitive to proteasome inhibitors [13], and some yeast cells that adapted to aneuploidy were found to contain mutations that derepress the UPS [6]. These data suggest that agents that inhibit PQC pathways should be more toxic to cancer cells than normal cells, and might be used to treat a broad variety of cancers. In the remainder of this review, I will refer to this idea as the ‘proteotoxic crisis’ approach to cancer therapy. Here, I will focus on targeting PQC pathways of the UPS as a means to induce proteotoxic crisis in cancer cells. Other reviews have focused specifically on targeting chaperones or autophagy as a means to treat cancer [11,12].

## Bortezomib validates the ‘proteotoxic crisis’ hypothesis but raises questions about its generality

The proteasome inhibitor bortezomib provided the first direct evidence that it is possible to inhibit the UPS in a manner that is lethal to at least some cancer cells while mostly sparing normal cells [14]. Before discussing bortezomib in detail, a primer on the structure and mechanism of the 26S proteasome is in order.

**Figure 2:**
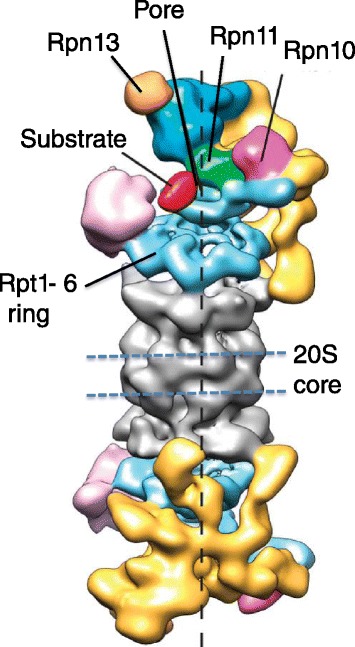
**Structure of the 26S proteasome.** The 20S core of the proteasome is shown in grey. One copy of each of the β1, β2, and β5 active sites is present within each of two seven-subunit rings (the positions of which are marked with dashed blue lines) and face towards the inside of the 20S chamber. Each end of the 20S core is capped with a 19S regulatory particle, shown in various colors. The Rpt1-6 ATPase ring that abuts the 20S core is shown in blue and Rpn11 in green. Rpn10 (purple) and Rpn13 (gold) are two intrinsic ubiquitin chain-binding receptors within the 26S proteasome. The pore in the ATPase ring through which the substrate passes is indicated. Electron density within the image that corresponds to substrate is shown in red. Adapted from [19].

The catalytic core of the proteasome is a 20S cylinder, the inside of which contains two copies each of the active sites β1, β2, and β5 (Figure 2) [15]. A second form of the proteasome, referred to as the immunoproteasome, is enriched in cells of the hematopoietic lineage and has a specialized function in immune cells, but an essentially analogous composition in which the β1, β2, and β5 sites are replaced by the closely related β1i, β2i, and β5i sites. The β5/β5i sites (also known as the chymotrypsin-like sites) are inhibited by bortezomib with high potency, whereas the β1 (caspase-like) sites have approximately 10-fold lower affinity and the β2 sites are not appreciably targeted under normal conditions [16-18]. Substrates enter the 20S cylinder through its ends, which are capped with structures referred to as 19S regulatory particles (RPs). A 20S cylinder capped at each end with a 19S RP is referred to as the 26S proteasome. Assembly of the 26S proteasome is enabled by pockets at the ends of the 20S cylinder into which are inserted short carboxy-terminal tails that emanate from a heterohexameric ring of Rpt1-6 subunits in the 19S RP. Degradation substrates are tethered to the 26S proteasome via their ubiquitin chain, which binds to one or more of a set of receptor proteins, some of which (for example, Rpn10 and Rpn13) are intrinsic to the 19S RP, while others (for example, hRad23, hPLIC) shuttle on and off.

It is thought that substrates are bound to the 26S proteasome in a manner that enables them to be grasped by the Rpt1-6 proteins, which are AAA ATPases that use the energy derived from ATP hydrolysis to unfold substrates, open the normally closed gate at the end of the 20S cylinder to admit substrate, and translocate the substrate through a pore in the center of the Rpt ring and into the internal chamber of the 20S cylinder. As substrate is being translocated through the Rpt ring, the Rpn11 subunit of the 19S RP, which is positioned immediately above the channel through the Rpt ring, scans for ubiquitin chains. Rpn11 is a protease that removes ubiquitin chains as the substrate translocates by, which is thought to prevent the chains from clogging up the entry channel into the proteasome.

Inhibition of 20S peptidase activity with bortezomib is highly cytotoxic to the plasma cell cancer multiple myeloma (MM) [20], and bortezomib has been an effective therapy for treating patients with this disease as well as mantle cell lymphoma (MCL) [21-23]. However, despite its considerable success as a therapy for MM and MCL, bortezomib has not been approved for treating other cancers. This is not for lack of effort: over 700 bortezomib trials have or are being run [24], including many in indications other than MM and MCL, in attempts to identify cancers that might respond favorably. This clinical experience is consistent with *in vitro* data: although brief exposure to proteasome inhibitors is highly cytotoxic to MM cells, it is not more cytotoxic to solid tumor cell lines than it is to non-transformed cells [25]. These data raise an obvious question - why aren’t proteasome inhibitors more broadly effective as cancer therapeutics - and pose a serious challenge to the generality of the proteotoxic crisis hypothesis.

Most attempts to explain why proteasome inhibitors work in MM and MCL but not in other cancers have focused on physiology. MM cells often have an elevated level of activity of the pro-survival transcription factor NF-κB [26]. Indeed, genomic and transcriptomic analyses have revealed recurrent alterations in MM cells that deregulate NF-κB [27-29]. Proteasome inhibitors block degradation of the NF-κB inhibitor IκB by the proteasome, thereby inhibiting inducible NF-κB activity [30]. This could explain why MM cells, particularly those accustomed to a high level of constitutive NF-κB activity, might be sensitive to bortezomib. However, this is unlikely to be the key mechanism of action, because an inhibitor of the IκB kinase IKK (which is also required for IκB degradation) is not as effective as bortezomib at killing MM cells [31]. Moreover, bortezomib does not downregulate NF-κB activity in primary MCL and MM cells or in MM xenografts [32,33].

An additional explanation for the sensitivity of MM cells to bortezomib is that they exhibit a lower threshold for induction of a lethal ‘unfolded protein response’ (UPR) [34]. The UPR is a homeostatic response that is mobilized by the presence of unfolded proteins in the lumen of the endoplasmic reticulum (ER) [35]. Under normal conditions, these unfolded proteins are retrotranslocated back to the cytosol, where they are degraded by the proteasome in a process known as ER-associated degradation (ERAD) [36]. However, when the burden of unfolded proteins in the ER lumen is high, activation of the UPR enables cells to cope with this problem by inhibiting protein synthesis to reduce the load on the ER while upregulating genes to enhance the biogenic capacity of the ER [35]. However, sustained UPR signaling can eventually commit a cell to apoptosis. Inhibition of the proteasome can activate an apoptotic UPR in myeloma cells [34], presumably by interfering with ERAD. MM plasma cells may be particularly prone to a cytotoxic UPR because of their physiological role in producing large quantities of antibody [37,38]. This perches the cells on the edge of proteotoxic crisis, and transient inhibition of the proteasome is the nudge that pushes them into the abyss. Exposure to proteasome inhibitors for as little as one hour can suffice to consign MM plasma cells to an apoptotic fate, whereas much higher levels of drug or longer exposures are normally required to induce cell death in solid tumor cells [25] and possibly in MM stem cells that are at an earlier developmental stage [38]. Notably, cancer cells isolated from MCL patients dosed with bortezomib do not exhibit a strong UPR but instead show evidence of NRF2 activation, suggesting that MM and MCL may respond to bortezomib therapy for different reasons [39]. A deeper understanding of why some MCL patients respond to bortezomib could suggest other cancers that may be prone to respond to proteasome inhibition in mono- or combination therapy.

If the proteotoxic crisis hypothesis is correct, it should be possible to identify cancer types and treatment regimes for which there is a favorable therapeutic index for killing tumor cells with mutation-riddled genomes while sparing normal cells. One place to start looking is in cancers that originate in secretory tissues, including neuroendocrine tumors in general and insulinoma in particular. However, the search need not be limited to ‘secretory’ tumors. Indeed, it was recently argued that the sensitivity of MM cells to proteasome inhibitors can be generalized to a simple metric comprising the rate of degradation of newly synthesized proteins (which is a crude measure of PQC) divided by the level of proteasome activity [40,41]. Using this metric, which is more broadly focused on PQC and does not necessarily invoke a unique role for ERAD or UPR, it may be possible to identify other cancers that are likely to be responsive to proteasome inhibition.

## Is the clinical action of bortezomib limited by pharmacokinetics?

**Figure 3:**
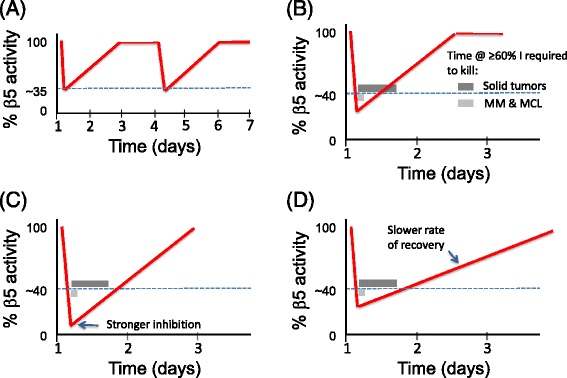
**The sawtooth pattern of β5 inhibition** ***in vivo*** **and its relationship to the kinetics of cancer cell death. (A)** A patient dosed with bortezomib at the beginning of day 1 experiences approximately 65% inhibition of β5 activity (shown as a red trace) in whole blood lysate. β5 activity recovers, and the patient is dosed again on day 4. **(B)** Zoom-in of (A) to emphasize β5’s pharmacodynamic response to a single dose. In this and the following examples, it is assumed that the kinetics of cancer cell death are a function of cell type and percentage inhibition. The example shown assumes that MM cells commit to cell death within a few hours when the proteasome is inhibited by more than 60% (>60% I), as signified by the time interval denoted by the light gray bar. On the other hand, solid tumor cells require much longer exposure (dark gray bar) to effect cell death at 60% inhibition. **(C)** Same as (B), except that a greater percentage inhibition of the proteasome is achieved. Even though the rate of recovery is the same as (B), it is suggested that solid tumor cells remain in the ‘kill zone’ below the dotted line sufficiently long to commit to apoptosis. Note that even though the time required for killing solid tumors (dark gray bar) is drawn the same as in (B), a greater percentage inhibition could reduce the time required to commit to apoptosis. **(D)** Same as (B), except the recovery curve has a shallower slope due to inhibition of new proteasome synthesis. In this hypothetical example, reducing the rate of recovery maintains proteasome inhibition in the ‘kill zone’ for a sufficiently long time to kill solid tumor cells.

To understand why bortezomib has not been an effective therapy for solid tumors and does not cure MM, it may be useful to consider not only the physiology, but also the pharmacology of proteasome inhibition. In general, although it is usually possible to kill solid tumor cells with bortezomib upon continuous exposure in plastic dishes without too much difficulty, it can be much more challenging to do so in mice. Among the numerous differences between these two venues, it may be particularly important to pay attention to pharmacokinetics. Following its injection, bortezomib is quickly cleared from human plasma [42]. In effect, injecting a human with bortezomib is akin to doing a pulse-chase experiment. Cells transiently exposed to bortezomib eventually recover proteasome activity, both because bortezomib dissociates from β5 (albeit slowly, with a t_1/2_ of 110 minutes [43]), and because inhibition of the proteasome sets in motion a homeostatic mechanism (described in more detail later in this review) that results in elevated transcription of genes that encode proteasome subunits [44,45]. Consequently, β5 inhibition follows a sawtooth pattern *in vivo* (Figure 3A), with rapid inhibition of its activity, followed by a slower recovery driven by drug dissociation with a possible contribution by new proteasome synthesis. As a result, cells experience maximum proteasome inhibition - the point of the sawtooth - for only a few hours [46].

When injected at its standard clinical dose (1.3 mg/m^2^), bortezomib elicits approximately 65% inhibition of β5 activity in whole blood lysate at the point of the sawtooth [47]. Importantly, biochemical studies suggest that inhibition of β5 is likely to be insufficient, and co-inhibition of the β1 site, which is 10-fold less sensitive to bortezomib than β5, is required to prevent protein breakdown [18]. Furthermore, whole blood β5 activity is more sensitive to bortezomib than solid tissues. At its maximum tolerated dose in mice, bortezomib inhibits β5 activity to approximately 90% in whole blood, but only approximately 75% in the adrenal gland and 50% in a myeloma cell xenograft [25]. A very recent study reported that following a 1 hour pulse treatment with 100 nM bortezomib (which exceeds by two-fold the concentration achieved following a subcutaneous dose), β5 activity was eliminated but proteasome-dependent proteolysis was inhibited by only 23 to 55% across seven MM cell lines and >70% cell death was observed in only one of the lines [41]. This study suggests that the degree of proteasome (as opposed to β5) inhibition elicited by bortezomib in tumor tissue *in vivo* - which has not been reported - is likely to be quite modest. Perhaps the depth and duration of proteasome suppression achieved *in vivo* is sufficient to kill MM plasma cells teetering on the edge of UPR-dependent apoptosis (Figure 3B), but not strong and long enough to kill MM stem cells and most solid tumor cells, including those that may have a heightened dependency on the UPS.

If this idea is correct, it suggests that it might be possible to expand the range of cancers in which proteasome inhibitor therapy is effective by increasing the extent of inhibition and reducing the rate at which proteasome activity recovers following inhibition. This idea was part of the motivation underlying the partnership that Craig Crews and I formed to co-found Proteolix. The Crews lab had discovered that the natural product epoxomicin is a covalent, irreversible inhibitor of the same β5 active site of the proteasome that is inhibited by bortezomib [48]. They then went on to develop YU101, which is a modified form of epoxomicin that is more specific for the β5 site than the parent molecule [49]. We reasoned that greater specificity might allow for a better tolerability profile than bortezomib and hence the potential to achieve stronger inhibition, whereas irreversibility would result in a longer duration of proteasome inhibition because the only way to recover activity would be to synthesize new proteasome. Proteolix modified YU101 to generate carfilzomib [25], which has emerged as a successful second-generation proteasome inhibitor drug. Carfilzomib, like bortezomib, is an injectable drug that is cleared rapidly from plasma [50]. Nevertheless, it has shown efficacy in relapsed and refractory MM patients [51] and has shown very promising activity in earlier stage myeloma patients in combination with lenalidomide plus dexamethasone [52]. By contrast, limited activity was observed in a phase I/II study that included four different solid tumor types [53]. However, as of October 2014 there are 63 open clinical trials involving carfilzomib listed on clinicaltrials.gov [54], including in kidney, prostate, lung, and ovarian cancer, and so the jury is still out. Interestingly, despite carfilzomib’s irreversibility, the rate of recovery of proteasome β5 activity in tissues other than whole blood following carfilzomib administration in mice is not very much slower than that observed with bortezomib [25]. Thus, synthesis of new proteasomes appears to be a powerful homeostatic mechanism that minimizes the duration of proteasome inhibition following a pulse of bortezomib or carfilzomib.

## Alternative paths to testing the hypothesis that cancer cells are vulnerable because of their heightened dependency on protein quality control

**Figure 4:**
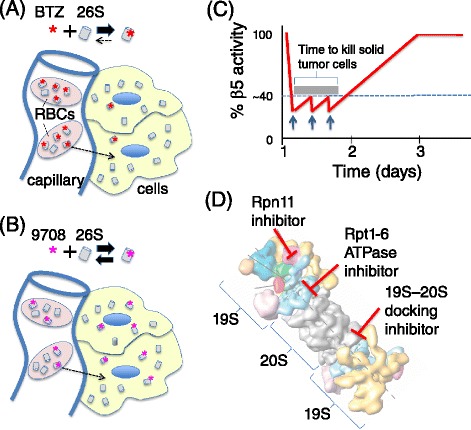
**Alternative strategies for testing the proteotoxic crisis hypothesis through proteasome inhibition. (A)** Bortezomib (BTZ; red asterisk) has a very slow off-rate from 26S proteasome (26S; gray cylinders). Coupled with the high concentrations of proteasomes in red blood cells (RBCs), this results in sequestration of most BTZ in the RBC compartment following intravenous injection. **(B)** MLN9708 (purple asterisk) dissociates from proteasome six-fold faster than BTZ, enabling better equilibration throughout the body and stronger inhibition of proteasome in tumors. **(C)** Hypothetical pharmacodynamic response of β5 activity (red trace) in a patient repeatedly dosed (blue arrows) with an oral proteasome inhibitor during the course of a single day. Repeat dosing may suffice to keep β5 activity in the ‘kill zone’ (in this example, >60% inhibition) for a sufficiently long time interval (denoted by dark gray bar) to kill solid tumor cells. **(D)** Alternative drug targets in the proteasome: the Rpt1-6 ATPase, Rpn11, and the pockets in 29S outer rings that serve as docking sites for Rpt ATPases in 19S regulatory particle.

In light of the experience with bortezomib and carfilzomib, is the proteotoxic crisis hypothesis likely to be generally applicable beyond MM and MCL? Given the challenge of establishing and maintaining a high level of PQC inhibition in tumors with bortezomib and carfilzomib, it is difficult to answer this question definitively. Answering it will require the development of new agents that allow for more potent and durable suppression of key PQC pathways in tumors. There are five strategies for moving forward. The first, which is somewhat counterintuitive, is to develop inhibitors with faster off-rates. It has recently been shown that ixazomib/MLN9708, an oral analog of bortezomib that is undergoing clinical evaluation, has a much more rapid off-rate from β5. Paradoxically, mice treated with this compound exhibit stronger inhibition of proteasome activity in xenografted tumors compared to mice treated with bortezomib [43]. This leads to a stronger anti-tumor effect, including responses in xenograft models that are minimally responsive to bortezomib. The authors propose that the high concentration of blood proteasome coupled with the extremely slow off-rate of bortezomib limits its access to peripheral tissues, which in turn may limit its effectiveness (Figure 4A). Presumably, the same issue may apply to carfilzomib. In effect, red blood cell proteasomes serve as a sponge, diminishing access of proteasome inhibitors to other tissues, including solid tumors. The high off-rate of ixazomib allows it to more efficiently equilibrate throughout the body (Figure 4B).

A second approach, which is also already underway, is to develop oral proteasome inhibitors that would allow for more flexibility in dosing. Ixazomib and oprozomib are oral analogs of bortezomib and carfilzomib, respectively, that are in mid- to late stage clinical development as therapies for MM. As noted above, current therapy with bortezomib and carfilzomib results in a sawtooth pattern of β5 inhibition (Figure 3A). Is it possible that maintaining a more constant level of inhibition for a longer duration via repetitive oral dosing (Figure 4C) might enable killing of solid tumor cells while sparing normal cells? It is difficult to test this hypothesis with injectable agents like bortezomib and carfilzomib because administration of the drug requires a visit to a doctor, but this approach could be accessible with oral agents, provided that they are tolerated by the gastrointestinal tract.

A third approach, related to the one described above, is to develop oral agents that target other aspects of proteasome function (Figure 4D). The proteasome is an extremely complex enzyme comprising multiple subcomplexes each of which has enzymatic sites that are essential for proteasome activity. There is a great deal of precedent indicating that agents that hit the same target but do so with a different molecular scaffold or by a different mechanism often have substantially different clinical properties. One example (among many) is the difference between vinca alkaloid antimicrotubule agents [55]. Two novel small molecules - b-AP15 and RA190 - that are proposed to kill cancer cells by inhibiting the proteasome have been reported. B-AP15 simultaneously inhibits the proteasome-associated deubiquitinating enzymes UchL5 and Usp14, whereas RA190 binds and inhibits the ubiquitin receptor subunit Rpn13 [56,57]. In addition, other targets, including Rpn11, the Rpt AAA ATPases, and the pockets in the 20S to which the Rpt subunits dock, should be drugable with small molecules. Although a high-throughput screening (HTS) assay that monitors assembly of 19S RPs with 20S cylinders has not been reported, an HTS assay for identifying inhibitors of Rpn11 and the Rpt enzymes was originally developed at Proteolix [58] and refined in my laboratory [59]. Implementation of our method allowed us to identify small molecules that are candidate Rpn11 inhibitors. It remains to be seen whether suitable molecules that inhibit the Rpt ATPases can be identified by this approach. A great deal of work needs to be done to develop clinical-grade molecules that inhibit other aspects of proteasome function, but the success of bortezomib and carfilzomib provides motivation for pursuing these targets.

The fourth approach is to combine proteasome inhibitors with other agents that influence PQC. This includes inhibitors of Hsp90 and HDAC6, as well as agents discussed in the next paragraph. To date, several efforts registered at clinicaltrials.gov have been initiated and/or completed. Whereas the data for Hsp90 plus proteasome inhibitor combinations have yet to yield an obvious clinical benefit, the HDAC inhibitor panobinostat in combination with bortezomib and dexamethasone yielded a statistically significant increase in progression-free survival compared to the control arm lacking panobinostat [60]. Ironically, the most successful combination with proteasome inhibitors has been the immunomodulatory agent lenalidomide, even though, superficially, it would appear that proteasome inhibitors and lenalidomide should counteract each other, because lenalidomide appears to work by activating degradation of the IKZF1 and IKZ3 transcription factors [61-63].

A fifth approach to addressing the proteotoxic crisis hypothesis is to identify other suitable targets in the UPS besides the 26S proteasome. Multiple efforts have been initiated in this direction. There have been many programs to generate inhibitors of E3 ubiquitin ligases and deubiquitinating enzymes, but these are not covered here because in all of these cases, the intention has been to prevent the degradation of tumor suppressor proteins (for example, p27 or p53) or accelerate the degradation of proto-oncoproteins (for example, Hdm2), and hence these efforts do not fit in the ‘proteotoxic crisis’ category elaborated on here.

In addition, there have been attempts to target more broadly acting components of the UPS, including the Nedd8 activating enzyme (NAE) [64]. Nedd8 is an ubiquitin-like protein that is conjugated to cullins following its activation by NAE. NAE-dependent conjugation of Nedd8 switches on the activity of cullin-RING ubiquitin ligases (CRLs), which number in the hundreds and play important roles in cell cycle control, signaling, and DNA damage, but have not been extensively linked to PQC. Thus, an NAE inhibitor is also not predicted to kill cancer cells by inducing proteotoxic crisis. The closely related ubiquitin-activating enzyme (UAE), on the other hand, is required for all ubiquitin-dependent degradation by the proteasome as well as non-degradative signaling by monoubiquitination, and thus its inhibition is likely to have very broad effects, including blockade of PQC and induction of proteotoxicity. Two different inhibitors of UAE have been reported - PYR41 [65] and the adenine sulfamate analog Compound I [66]. PYR-41 blocks accumulation of ubiquitin conjugates, promotes accumulation of p53, and preferentially kills transformed cells that express p53. However, the specificity of this molecule for UAE versus other cysteine-based enzymes was not evaluated in depth. Meanwhile, Millennium Pharmaceuticals’ Compound I blocks formation of E2-ubiquitin thioesters and polyubiquitin conjugates in cells, but its effects on cell viability were not reported. However, clinicaltrials.gov lists an active phase 1 trial sponsored by Millennium for the ubiquitin-activating enzyme inhibitor MLN7243 [67]. Because there are no publications yet that name this molecule, it is not known how it relates to Compound I.

Other targets that have been pursued in the broader arena of PQC include the transmembrane signaling enzymes IRE1 [68-70] and PERK [71-73]. Neither of these proteins is a UPS component *per se*, but I discuss them here briefly because they are involved in a regulatory response that is intimately connected to the UPS. Both PERK and IRE1 are transmembrane proteins of the ER membrane that contain cytosolic protein kinase domains. IRE1 also contains a cytosolic endoribonuclease activity. Both of these proteins sense misfolded proteins in the ER and employ their kinase (PERK) or nuclease (IRE1) domains to signal the presence of unfolded proteins in the ER to the cytosol and nucleus. This results in induction of the UPR, leading to downregulation of general translation and upregulation of proteins that increase the biosynthetic capacity of the ER. Inhibition of PERK or IRE1 thus has potential to induce a proteotoxic crisis by preventing the UPR in cancers such as MM that may rely on the UPR for survival. Indeed, both PERK [73] and IRE1 [70] inhibitors are cytotoxic to cancer cells and have shown activity in multiple myeloma xenograft models. However, the PERK inhibitor exhibited pancreatic toxicity, which may complicate its clinical development. There has yet to be a human clinical trial that targets either enzyme.

Several years ago, my laboratory embarked on the path of identifying a new target in PQC. The criteria we set forth was that the ideal target should be: (i) drugable (that is, an enzyme); (ii) a key player in PQC; and (iii) mutated, amplified, hyperactivated, or overexpressed in some cancers, consistent with the idea that its activity contributes to the cancer lifestyle. To this, we added the optional criterion that an optimal target would be required for both NF-κB activation and ERAD. In surveying the UPS landscape we settled on the AAA ATPase p97, also known as valosin-containing protein (VCP). At that time, p97 was well-known to be required for ERAD [74] and had been linked to NF-κB regulation by co-immunoprecipitation studies [75]. Recent functional studies have confirmed the significance of the physical interactions [76]. p97 was also known to be overexpressed in multiple cancers [77-83], pointing to a possible addiction [84].

## p97: a key player in protein quality control

**Figure 5:**
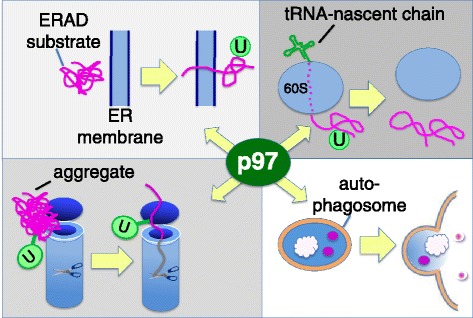
**Roles of p97 in protein quality control.** Upper left: p97 extracts unfolded or misassembled secretory and membrane proteins from the ER. Concomitant with extraction, substrates are conjugated with ubiquitin (green circle with U). Upper right: ribosomes that stall during translation are disassembled into 40S +60S by an upstream factor. The ubiquitin-conjugated nascent chain remains attached to tRNA and passes through the exit tunnel (dotted segment). p97 recognizes these complexes and releases the nascent chain from the ribosome. Lower left and right: protein and protein-RNA aggregates require p97 for metabolism. p97 may either disassemble aggregates so that the proteasome can degrade them (lower left), or the aggregates can be packaged into autophagosomes (lower right) for delivery to the lysosome. p97 is required for an undetermined step in autophagosome maturation.

As described at the outset of this article, substrates modified with ubiquitin chains are bound and degraded by the 26S proteasome. In many cases, the proteasome does not require assistance [85]. However, there are some ubiquitin-conjugated substrates that the 26S proteasome is unable to degrade without additional help from p97. p97 is a homohexamer that associates with different adaptors to promote degradation of a subset of UPS targets. In particular, p97 activity has been linked to PQC pathways (Figure 5). p97 functions downstream of ubiquitin ligases and in conjunction with the 26S proteasome, helping to extract ubiquitinated substrates from cellular structures (Figure 5 upper left and right) and/or unfold them (Figure 5 lower left) so that they can be threaded into the proteasome for degradation [86,87]. The requirement for p97 in the UPS is best understood in the context of ERAD. p97 promotes retrotranslocation of proteasome substrates across the ER membrane so that they can gain access to the proteasome [74]. Thus, in the absence of p97 activity, ERAD substrates accumulate in the ER. It is thought that all ERAD substrates depend on p97 for their degradation [88]. However, a great deal remains unknown about p97. For example, it is not known why some nuclear and cytosolic substrates of the UPS depend on p97 for their degradation and others do not. In some cases like ERAD and degradation of RNA polymerase II stalled at sites of DNA damage [89], p97 is required to extract the substrate from a larger biological structure that may impede access of the 26S proteasome. In other cases such as the model substrate ubiquitin-GFP, p97 may initiate unfolding to reveal an unstructured region that can be grasped by the 26S proteasome [90]. I would like to suggest that substrate dependence on p97 defines a continuum, with the degree of dependence inversely proportional to the statistical probability that the 26S proteasome ATPases are sufficient to capture, unfold and translocate a substrate into the 20S cavity before it dissociates. An additional mystery that remains to be solved is, how does p97 recognize its nuclear and cytosolic substrates and process them for degradation?

Despite the relative paucity of insight into the mechanism of p97 function, considerable progress has been made recently in linking p97 to various substrates and quality control processes within the UPS. In addition to ERAD (Figure 5 upper left), it has been shown that p97 participates in multiple PQC pathways, including ribosome-associated degradation (RAD; Figure 5 upper right) of peptides produced from defective mRNAs [91-93] and clearance of ribonucleoprotein stress granules [94]. The connection between p97 and autophagy [95-97] may be of particular significance, because cells can adapt to genetic suppression of proteasome function by upregulating autophagy [98] - an option that might not be available in a cell exposed to a p97 inhibitor. Taken together, these data implicate p97 as a critical player central to protein homeostasis. In addition, p97 has been linked to numerous other degradation pathways in the UPS. For a more thorough discussion of the PQC and non-PQC functions of p97, please consult [86,87].

## Identification of p97 ATPase inhibitors

**Figure 6:**
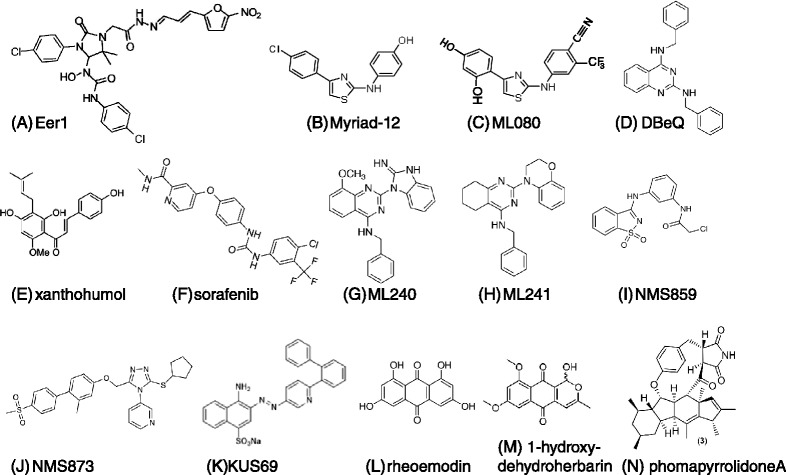
**Structures of p97 inhibitors.** The inhibitors are as indicated in the figure. Inhibitors are listed in order of their first report in the literature. Myriad-19 is similar to Myriad-12 except that it lacks the chlorine atom. ML080 behaves essentially the same as the Myriad compounds. KUS69 is the most potent of a series of five structurally related compounds.

Cancer cells rapidly activate caspase and undergo cell death upon depletion of p97 [99,100]. However, primary rat hepatocytes [101] and mouse skeletal muscle cells [102] do not undergo apoptosis upon p97 depletion, raising the possibility that p97 inhibitors might be more cytotoxic to cancer cells than normal cells. To address the potential of p97 as a drug target in oncology, we and others set out to identify potent and specific inhibitors of p97 ATPase activity (Figure 6). So far, the inhibitors that exhibit the best combination of biochemical and cell-based potency and specificity are ML240 and ML241 [103] (Figure 6G,H), which are based on the quinazoline scaffold DBeQ [100] (Figure 6D), and NMS873 [99] (Figure 6J). These are described in more detail below. In addition a number of other inhibitors have been reported, including Eer1 [104] (Figure 6A), 2-anilino-4-aryl-1,3-thiazoles (Figure 6B,C) [105,106], xanthohumol [107] (Figure 6E), the tyrosine kinase inhibitor sorafenib [108] (Figure 6F), the covalent inhibitor NMS859 [99] (Figure 6I), KUS69 [109] (the most potent of five closely related KUS compounds), rheoemodin (Figure 6L), 1-hydroxydehydroherbarin (Figure 6M), phomapyrrolidone A [110] (Figure 6N), and Syk inhibitor III [111].

To screen for p97 inhibitors, we developed an assay to monitor the action of p97 in cells by exploiting the observation that proteins fused to the carboxyl terminus of ubiquitin are degraded by the ‘ubiquitin fusion degradation’ (UFD) pathway, of which p97 is a component [112]. Our assay relies on accumulation of Ub^G76V^-GFP using the rapidly reversible proteasome inhibitor MG132 [113]. MG132 is then removed and the decay of the pre-accumulated Ub^G76V^-GFP signal is monitored in the presence of the protein synthesis inhibitor cycloheximide. To evaluate specificity, we monitor degradation of an ODD-luciferase chimera (ODD is the oxygen-dependent degradation domain from HIF-1α) [114]. p97 is not required for ODD-luciferase degradation, and hence p97 inhibitors stabilize Ub^G76V^-GFP but not ODD-luciferase [111]. By this criterion, DBeQ was identified as a selective p97 inhibitor and is the first selective inhibitor of an AAA ATPase activity. Structure-activity relationships analysis of DBeQ identified the more potent derviatives ML240 and ML241 (Figure 6G,H) [103]. Whereas all three compounds stabilize Ub^G76V^-GFP, cause accumulation of ubiquitin conjugates, and inhibit degradation of an ERAD substrate, DBeQ and ML240 also cause accumulation of LC3-II (indicative of a block to autophagy) and induce rapid cell death (with modest selectivity for transformed cells), whereas ML241 does not. The basis for this different behavior remains unclear.

NMS859 and NMS873 (Figure 6I,J), arose from a high-throughput screen for p97 ATPase inhibitors followed by a structure-activity relationships analysis [99,115]. Of particular interest is NMS873, which is a reversible, allosteric inhibitor of p97 ATPase. It is the most potent p97 ATPase inhibitor reported to date, with an IC_50_ of approximately 30 nM. Similar to DBeQ, NMS873 impinges on both the UPS and autophagy, and is cytotoxic across a broad range of cancer cell lines. Interestingly, unlike proteasome inhibitors, it is not more cytotoxic to MM cells than to other cancer cells. Its relative cytotoxicity in non-transformed cells was not reported.

## p97 inhibitors and the proteasome recovery pathway

**Figure 7:**
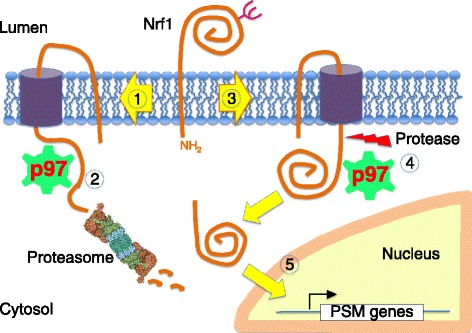
**Mechanism of Nrf1 activation.** Upon completion of synthesis, Nrf1 is rapidly directed into the retrotranslocation pathway (1). p97 extracts Nrf1 from the ER, and it is then fed to the proteasome (2). Because of the tight coupling between synthesis, retrotranslocation, and degradation, there is very little accumulation of Nrf1 at steady-state. In cells that are deficient in proteasome activity (3), retrotranslocation and degradation of Nrf1 become kinetically uncoupled. Accumulation of Nrf1 on the cytosolic side of the membrane renders it susceptible to cleavage by an unknown protease (5), which releases a soluble 110 kDa fragment that translocates to the nucleus and activates transcription of genes that encode proteasome subunits. PSM: proteasome components.

When the proteasome is inhibited, cells respond by upregulating the transcription of genes that encode proteasome subunits. This regulation depends on the transcription factor Nrf1/NFE2L1 [116,117]. We and others [118-120] have investigated the mechanism by which Nrf1 is activated (Figure 7). Nrf1 is initially synthesized as a type II transmembrane protein of the ER, such that a very small segment of the amino terminus faces the cytosol, and the bulk of the protein is in the ER lumen. However, this species is a very short-lived intermediate, and the carboxy-terminal domain of Nrf1 is quickly retrotranslocated to the cytosolic face of the ER in a manner that depends upon p97. Under normal circumstances, retrotranslocation of Nrf1 is coupled to its degradation by the proteasome, so that active Nrf1 does not accumulate. However, if the proteasome is inhibited, the retrotranslocated Nrf1 is cleaved after Trp103 to release a carboxy-terminal fragment, p110, which travels to the nucleus and activates gene expression [121]. When p97 is inhibited, Nrf1 is not retrotranslocated, and the carboxy-terminal domain, which contains the sequences that mediate gene activation, remains in the ER lumen. Consequently, simultaneous inhibition of p97 and the proteasome prevents formation of p110 and activation of proteasome gene expression that normally follows inhibition of the proteasome. This could explain a recent observation that proteasome and p97 inhibitors exhibit synergistic activity towards MM cells *in vitro* [122]. Thus, it may be possible to control the rate of recovery of proteasome activity by combining an irreversible proteasome inhibitor like carfilzomib with a p97 inhibitor. The protease that cleaves after Trp103 is in dispute [118,120]. Inhibition of this processing step is also a potentially interesting target, because a non-cleavable mutant of Nrf1 cannot be activated upon inhibition of the proteasome [118].

## PQC inhibitors and cancer therapy

To explore further the clinical potential of ML240 and ML241, as well as a small-molecule scaffold that inhibits both Rpn11 and the Csn5 subunit of the COP9-signalosome complex (not discussed here), my partners and I launched Cleave Biosciences. Cleave has made rapid progress on the ML240 scaffold, and the derivative CB-5083 recently entered human phase I trials in MM and solid tumors [123].

It remains to be seen whether cancer cells in their natural environment are more sensitive than normal cells to the proteotoxicity induced by UAE and p97 inhibitors, and whether aggravation of proteotoxic stress in cancer can be achieved with an acceptable side effect profile. With UAE and p97 inhibitors now in the clinic, we should not have to wait much longer for an answer.

## Prospects for establishing the proteotoxic principle in tumor therapy

The proteotoxic crisis hypothesis suggests the attractive prospect that it may be possible to attack a broad range of human cancers by taking advantage of their presumed heightened dependence on PQC pathways. This heightened dependency is predicted to arise from the very mutations and genomic instabilities that fuel development of the cancer in the first place. The clinical experience to date with the proteasome inhibitor bortezomib on the one hand suggests that the proteotoxic crisis hypothesis may apply to at least some cancers, but on the other hand may not be broadly applicable. However, the limited efficacy of bortezomib in solid tumors may be due to the pharmacology of the existing proteasome inhibitors and the existence of a cellular homeostatic mechanism that enables a compensatory response to proteasome inhibition, rather than a problem with the proteotoxic crisis hypothesis *per se*. New approaches to inhibiting the proteasome or other UPS targets like UAE and p97 may provide a more salient test of the hypothesis that cancer cells, broadly speaking, are more dependent on PQC pathways than normal cells and thus should be selectively vulnerable to inhibition of PQC.

## References

[1] Hershko A, Ciechanover A (1998). The ubiquitin system. Annu Rev Biochem.

[2] Balch WE, Morimoto RI, Dillin A, Kelly JW (2008). Adapting proteostasis for disease intervention. Science.

[3] Vogelstein B, Papadopoulos N, Velculescu VE, Zhou S, Diaz LA, Kinzler KW (2013). Cancer genome landscapes. Science.

[4] Weaver BA, Cleveland DW (2006). Does aneuploidy cause cancer?. Curr Opin Cell Biol.

[5] Williams BR, Prabhu VR, Hunter KE, Glazier CM, Whittaker CA, Housman DE, Amon A (2008). Aneuploidy affects proliferation and spontaneous immortalization in mammalian cells. Science.

[6] Torres EM, Dephoure N, Panneerselvam A, Tucker CM, Whittaker CA, Gygi SP, Dunham MJ, Amon A (2010). Identification of aneuploidy-tolerating mutations. Cell.

[7] Warner JR, Mitra G, Schwindinger WF, Studeny M, Fried HM (1985). Saccharomyces cerevisiae coordinates accumulation of yeast ribosomal proteins by modulating mRNA splicing, translational initiation, and protein turnover. Mol Cell Biol.

[8] Dephoure N, Hwang S, O'Sullivan C, Dodgson SE, Gygi SP, Amon A, Torres EM (2014). Quantitative proteomic analysis reveals posttranslational responses to aneuploidy in yeast. eLife.

[9] Williams BR, Amon A (2009). Aneuploidy: cancer's fatal flaw?. Cancer Res.

[10] Luo J, Solimini NL, Elledge SJ (2009). Principles of cancer therapy: oncogene and non-oncogene addiction. Cell.

[11] Whitesell L, Lindquist SL (2005). HSP90 and the chaperoning of cancer. Nat Rev Cancer.

[12] Guo JY, Xia B, White E (2013). Autophagy-mediated tumor promotion. Cell.

[13] Torres EM, Sokolsky T, Tucker CM, Chan LY, Boselli M, Dunham MJ, Amon A (2007). Effects of aneuploidy on cellular physiology and cell division in haploid yeast. Science.

[14] Adams J (2004). The proteasome: a suitable antineoplastic target. Nat Rev Cancer.

[15] Finley D (2009). Recognition and processing of ubiquitin-protein conjugates by the proteasome. Annu Rev Biochem.

[16] Berkers CR, Verdoes M, Lichtman E, Fiebiger E, Kessler BM, Anderson KC, Ploegh HL, Ovaa H, Galardy PJ (2005). Activity probe for in vivo profiling of the specificity of proteasome inhibitor bortezomib. Nat Methods.

[17] Altun M, Galardy PJ, Shringarpure R, Hideshima T, LeBlanc R, Anderson KC, Ploegh HL, Kessler BM (2005). Effects of PS-341 on the activity and composition of proteasomes in multiple myeloma cells. Cancer Res.

[18] Kisselev AF, Callard A, Goldberg AL (2006). Importance of the different proteolytic sites of the proteasome and the efficacy of inhibitors varies with the protein substrate. J Biol Chem.

[19] Matyskiela ME, Lander GC, Martin A (2013). Conformational switching of the 26S proteasome enables substrate degradation. Nat Struct Mol Biol.

[20] Hideshima T, Bradner JE, Wong J, Chauhan D, Richardson P, Schreiber SL, Anderson KC (2005). Small-molecule inhibition of proteasome and aggresome function induces synergistic antitumor activity in multiple myeloma. Proc Natl Acad Sci U S A.

[21] Goy A, Younes A, McLaughlin P, Pro B, Romaguera JE, Hagemeister F, Fayad L, Dang NH, Samaniego F, Wang M, Broglio K, Samuels B, Gilles F, Sarris AH, Hart S, Trehu E, Schenkein D, Cabanillas F, Rodriguez AM (2005). Phase II study of proteasome inhibitor bortezomib in relapsed or refractory B-cell non-Hodgkin's lymphoma. J Clin Oncol.

[22] O'Connor OA, Wright J, Moskowitz C, Muzzy J, MacGregor-Cortelli B, Stubblefield M, Straus D, Portlock C, Hamlin P, Choi E, Dumetrescu O, Esseltine D, Trehu E, Adams J, Schenkein D, Zelenetz AD (2005). Phase II clinical experience with the novel proteasome inhibitor bortezomib in patients with indolent non-Hodgkin's lymphoma and mantle cell lymphoma. J Clin Oncol.

[23] Richardson PG, Sonneveld P, Schuster MW, Irwin D, Stadtmauer EA, Facon T, Harousseau JL, Ben-Yehuda D, Lonial S, Goldschmidt H, Reece D, San-Miguel JF, Bladé J, Boccadoro M, Cavenagh J, Dalton WS, Boral AL, Esseltine DL, Porter JB, Schenkein D, Anderson KC, Assessment of Proteasome Inhibition for Extending Remissions (APEX) Investigator (2005). Bortezomib or high-dose dexamethasone for relapsed multiple myeloma. N Engl J Med.

[24] **ClinicalTrials.gov, search term "bortezomib".** In [http://clinicaltrials.gov/ct2/results?term=bortezomib&Search=Search]

[25] Demo SD, Kirk CJ, Aujay MA, Buchholz TJ, Dajee M, Ho MN, Jiang J, Laidig GJ, Lewis ER, Parlati F, Shenk KD, Smyth MS, Sun CM, Vallone MK, Woo TM, Molineaux CJ, Bennett MK (2007). Antitumor activity of PR-171, a novel irreversible inhibitor of the proteasome. Cancer Res.

[26] Ni H, Ergin M, Huang Q, Qin JZ, Amin HM, Martinez RL, Saeed S, Barton K, Alkan S (2001). Analysis of expression of nuclear factor kappa B (NF-kappa B) in multiple myeloma: downregulation of NF-kappa B induces apoptosis. Br J Haematol.

[27] Annunziata CM, Davis RE, Demchenko Y, Bellamy W, Gabrea A, Zhan F, Lenz G, Hanamura I, Wright G, Xiao W, Dave S, Hurt EM, Tan B, Zhao H, Stephens O, Santra M, Williams DR, Dang L, Barlogie B, Shaughnessy JD, Kuehl WM, Staudt LM (2007). Frequent engagement of the classical and alternative NF-kappaB pathways by diverse genetic abnormalities in multiple myeloma. Cancer Cell.

[28] Keats JJ, Fonseca R, Chesi M, Schop R, Baker A, Chng WJ, Van Wier S, Tiedemann R, Shi CX, Sebag M, Braggio E, Henry T, Zhu YX, Fogle H, Price-Troska T, Ahmann G, Mancini C, Brents LA, Kumar S, Greipp P, Dispenzieri A, Bryant B, Mulligan G, Bruhn L, Barrett M, Valdez R, Trent J, Stewart AK, Carpten J, Bergsagel PL (2007). Promiscuous mutations activate the noncanonical NF-kappaB pathway in multiple myeloma. Cancer Cell.

[29] Lohr JG, Stojanov P, Carter SL, Cruz-Gordillo P, Lawrence MS, Auclair D, Sougnez C, Knoechel B, Gould J, Saksena G, Cibulskis K, McKenna A, Chapman MA, Straussman R, Levy J, Perkins LM, Keats JJ, Schumacher SE, Getz G, Rosenberg M, Golub TR, Multiple Myeloma Research Consortium (2014). Widespread genetic heterogeneity in multiple myeloma: implications for targeted therapy. Cancer Cell.

[30] Palombella VJ, Rando OJ, Goldberg AL, Maniatis T (1994). The ubiquitin-proteasome pathway is required for processing the NF-kappa B1 precursor protein and the activation of NF-kappa B. Cell.

[31] Hideshima T, Chauhan D, Richardson P, Mitsiades C, Mitsiades N, Hayashi T, Munshi N, Dang L, Castro A, Palombella V, Adams J, Anderson KC (2002). NF-kappa B as a therapeutic target in multiple myeloma. J Biol Chem.

[32] Hideshima T, Ikeda H, Chauhan D, Okawa Y, Raje N, Podar K, Mitsiades C, Munshi NC, Richardson PG, Carrasco RD, Anderson KC (2009). Bortezomib induces canonical nuclear factor-kappaB activation in multiple myeloma cells. Blood.

[33] Yang DT, Young KH, Kahl BS, Markovina S, Miyamoto S (2008). Prevalence of bortezomib-resistant constitutive NF-kappaB activity in mantle cell lymphoma. Mol Cancer.

[34] Obeng EA, Carlson LM, Gutman DM, Harrington WJ, Lee KP, Boise LH (2006). Proteasome inhibitors induce a terminal unfolded protein response in multiple myeloma cells. Blood.

[35] Walter P, Ron D (2011). The unfolded protein response: from stress pathway to homeostatic regulation. Science.

[36] Smith MH, Ploegh HL, Weissman JS (2011). Road to ruin: targeting proteins for degradation in the endoplasmic reticulum. Science.

[37] Meister S, Schubert U, Neubert K, Herrmann K, Burger R, Gramatzki M, Hahn S, Schreiber S, Wilhelm S, Herrmann M, Jäck HM, Voll RE (2007). Extensive immunoglobulin production sensitizes myeloma cells for proteasome inhibition. Cancer Res.

[38] Leung-Hagesteijn C, Erdmann N, Cheung G, Keats JJ, Stewart AK, Reece DE, Chung KC, Tiedemann RE (2013). Xbp1s-negative tumor B cells and pre-plasmablasts mediate therapeutic proteasome inhibitor resistance in multiple myeloma. Cancer Cell.

[39] Weniger MA, Rizzatti EG, Pérez-Galán P, Liu D, Wang Q, Munson PJ, Raghavachari N, White T, Tweito MM, Dunleavy K, Ye Y, Wilson WH, Wiestner A (2011). Treatment-induced oxidative stress and cellular antioxidant capacity determine response to bortezomib in mantle cell lymphoma. Clin Cancer Res.

[40] Cenci S, Oliva L, Cerruti F, Milan E, Bianchi G, Raule M, Mezghrani A, Pasqualetto E, Sitia R, Cascio P (2012). Pivotal Advance: protein synthesis modulates responsiveness of differentiating and malignant plasma cells to proteasome inhibitors. J Leukocyte Biol.

[41] Shabaneh TB, Downey SL, Goddard AL, Screen M, Lucas MM, Eastman A, Kisselev AF (2013). Molecular basis of differential sensitivity of myeloma cells to clinically relevant bolus treatment with bortezomib. PLoS One.

[42] Papandreou CN, Daliani DD, Nix D, Yang H, Madden T, Wang X, Pien CS, Millikan RE, Tu SM, Pagliaro L, Kim J, Adams J, Elliott P, Esseltine D, Petrusich A, Dieringer P, Perez C, Logothetis CJ (2004). Phase I trial of the proteasome inhibitor bortezomib in patients with advanced solid tumors with observations in androgen-independent prostate cancer. J Clin Oncol.

[43] Kupperman E, Lee EC, Cao Y, Bannerman B, Fitzgerald M, Berger A, Yu J, Yang Y, Hales P, Bruzzese F, Liu J, Blank J, Garcia K, Tsu C, Dick L, Fleming P, Yu L, Manfredi M, Rolfe M, Bolen J (2010). Evaluation of the proteasome inhibitor MLN9708 in preclinical models of human cancer. Cancer Res.

[44] Mitsiades N, Mitsiades CS, Poulaki V, Chauhan D, Fanourakis G, Gu X, Bailey C, Joseph M, Libermann TA, Treon SP, Munshi NC, Richardson PG, Hideshima T, Anderson KC (2002). Molecular sequelae of proteasome inhibition in human multiple myeloma cells. Proc Natl Acad Sci U S A.

[45] Meiners S, Heyken D, Weller A, Ludwig A, Stangl K, Kloetzel PM, Kruger E (2003). Inhibition of proteasome activity induces concerted expression of proteasome genes and de novo formation of Mammalian proteasomes. J Biol Chem.

[46] Suzuki E, Demo S, Deu E, Keats J, Arastu-Kapur S, Bergsagel PL, Bennett MK, Kirk CJ (2011). Molecular mechanisms of bortezomib resistant adenocarcinoma cells. PLoS One.

[47] Aghajanian C, Soignet S, Dizon DS, Pien CS, Adams J, Elliott PJ, Sabbatini P, Miller V, Hensley ML, Pezzulli S, Canales C, Daud A, Spriggs DR (2002). A phase I trial of the novel proteasome inhibitor PS341 in advanced solid tumor malignancies. Clin Cancer Res.

[48] Meng L, Mohan R, Kwok BH, Elofsson M, Sin N, Crews CM (1999). Epoxomicin, a potent and selective proteasome inhibitor, exhibits in vivo antiinflammatory activity. Proc Natl Acad Sci U S A.

[49] Myung J, Kim KB, Lindsten K, Dantuma NP, Crews CM (2001). Lack of proteasome active site allostery as revealed by subunit-specific inhibitors. Mol Cell.

[50] O'Connor OA, Stewart AK, Vallone M, Molineaux CJ, Kunkel LA, Gerecitano JF, Orlowski RZ (2009). A phase 1 dose escalation study of the safety and pharmacokinetics of the novel proteasome inhibitor carfilzomib (PR-171) in patients with hematologic malignancies. Clin Cancer Res.

[51] Siegel DS, Martin T, Wang M, Vij R, Jakubowiak AJ, Lonial S, Trudel S, Kukreti V, Bahlis N, Alsina M, Chanan-Khan A, Buadi F, Reu FJ, Somlo G, Zonder J, Song K, Stewart AK, Stadtmauer E, Kunkel L, Wear S, Wong AF, Orlowski RZ, Jagannath S (2012). A phase 2 study of single-agent carfilzomib (PX-171-003-A1) in patients with relapsed and refractory multiple myeloma. Blood.

[52] Wang M, Martin T, Bensinger W, Alsina M, Siegel DS, Kavalerchik E, Huang M, Orlowski RZ, Niesvizky R (2013). Phase 2 dose-expansion study (PX-171-006) of carfilzomib, lenalidomide, and low-dose dexamethasone in relapsed or progressive multiple myeloma. Blood.

[53] Papadopoulos KP, Burris HA, Gordon M, Lee P, Sausville EA, Rosen PJ, Patnaik A, Cutler RE, Wang Z, Lee S, Jones SF, Infante JR (2013). A phase I/II study of carfilzomib 2-10-min infusion in patients with advanced solid tumors. Cancer Chemother Pharmacol.

[54] **ClinicalTrials.gov, search term, "carfilzomib".** In [http://clinicaltrials.gov/ct2/results?term=carfilzomib&recr=Open]

[55] Rowinsky E, Kuffe DW, Pollock RE, Weischselbaum RR (2003). The Vinca Alkaloids. Holland-Frei Cancer Medicine.

[56] Anchoori RK, Karanam B, Peng S, Wang JW, Jiang R, Tanno T, Orlowski RZ, Matsui W, Zhao M, Rudek MA, Hung CF, Chen X, Walters KJ, Roden RB (2013). A bis-benzylidine piperidone targeting proteasome ubiquitin receptor RPN13/ADRM1 as a therapy for cancer. Cancer Cell.

[57] Tian Z, D'Arcy P, Wang X, Ray A, Tai YT, Hu Y, Carrasco RD, Richardson P, Linder S, Chauhan D, Anderson KC (2014). A novel small molecule inhibitor of deubiquitylating enzyme USP14 and UCHL5 induces apoptosis in multiple myeloma and overcomes bortezomib resistance. Blood.

[58] Parlati F, Aujay M, Bennett MK: **Substrate for Rpn11 enzymatic activity.** In United States: Proteolix; 2010.

[59] PubChem: **Summary assay for small molecule inhibitors of Rpn11 in a Fluorescent Polarization assay.** In [http://pubchem.ncbi.nlm.nih.gov/assay/assay.cgi?aid=588509&loc=ea_ras]

[60] Richardson PG, Hungria VTM, Yoon SS, Beksac M, Dimopoulos MA, Elghandour A, Jedrzejczak WW, Guenther A, Nakorn TN, Siritanaratkul N, Schlossman RL, Hou J, Moreau P, Lonial S, Lee JH, Einsele H, Sopala M, Bengoudifa B-R, Corrado C, San-Miguel JF (2014). Panorama 1: a randomized, double-blind, phase 3 study of panobinostat or placebo plus bortezomib and dexamethasone in relapsed or relapsed and refractory multiple myeloma. J Clin Oncol.

[61] Krönke J, Udeshi ND, Narla A, Grauman P, Hurst SN, McConkey M, Svinkina T, Heckl D, Comer E, Li X, Ciarlo C, Hartman E, Munshi N, Schenone M, Schreiber SL, Carr SA, Ebert BL (2014). Lenalidomide causes selective degradation of IKZF1 and IKZF3 in multiple myeloma cells. Science.

[62] Lu G, Middleton RE, Sun H, Naniong M, Ott CJ, Mitsiades CS, Wong KK, Bradner JE, Kaelin WG (2014). The myeloma drug lenalidomide promotes the cereblon-dependent destruction of Ikaros proteins. Science.

[63] Gandhi AK, Kang J, Havens CG, Conklin T, Ning Y, Wu L, Ito T, Ando H, Waldman MF, Thakurta A, Klippel A, Handa H, Daniel TO, Schafer PH, Chopra R (2014). Immunomodulatory agents lenalidomide and pomalidomide co-stimulate T cells by inducing degradation of T cell repressors Ikaros and Aiolos via modulation of the E3 ubiquitin ligase complex CRL4(CRBN.). Br J Haematol.

[64] Soucy TA, Smith PG, Milhollen MA, Berger AJ, Gavin JM, Adhikari S, Brownell JE, Burke KE, Cardin DP, Cullis CA (2009). An inhibitor of NEDD8-activating enzyme as a novel approach to treat cancer. Nature.

[65] Yang Y, Kitagaki J, Dai RM, Tsai YC, Lorick KL, Ludwig RL, Pierre SA, Jensen JP, Davydov IV, Oberoi P, Li CC, Kenten JH, Beutler JA, Vousden KH, Weissman AM (2007). Inhibitors of ubiquitin-activating enzyme (E1), a new class of potential cancer therapeutics. Cancer Res.

[66] Chen JJ, Tsu CA, Gavin JM, Milhollen MA, Bruzzese FJ, Mallender WD, Sintchak MD, Bump NJ, Yang X, Ma J, Loke HK, Xu Q, Li P, Bence NF, Brownell JE, Dick LR (2011). Mechanistic studies of substrate-assisted inhibition of ubiquitin-activating enzyme by adenosine sulfamate analogues. J Biol Chem.

[67] **ClinicalTrials.gov, search term, "MLN7243".** In [http://clinicaltrials.gov/ct2/show/NCT02045095?term=mln7243&rank=1]

[68] Volkmann K, Lucas JL, Vuga D, Wang X, Brumm D, Stiles C, Kriebel D, Der-Sarkissian A, Krishnan K, Schweitzer C, Liu Z, Malyankar UM, Chiovitti D, Canny M, Durocher D, Sicheri F, Patterson JB (2011). Potent and selective inhibitors of the inositol-requiring enzyme 1 endoribonuclease. J Biol Chem.

[69] Cross BC, Bond PJ, Sadowski PG, Jha BK, Zak J, Goodman JM, Silverman RH, Neubert TA, Baxendale IR, Ron D, Harding HP (2012). The molecular basis for selective inhibition of unconventional mRNA splicing by an IRE1-binding small molecule. Proc Natl Acad Sci U S A.

[70] Papandreou I, Denko NC, Olson M, Van Melckebeke H, Lust S, Tam A, Solow-Cordero DE, Bouley DM, Offner F, Niwa M, Koong AC (2011). Identification of an Ire1alpha endonuclease specific inhibitor with cytotoxic activity against human multiple myeloma. Blood.

[71] Wang H, Blais J, Ron D, Cardozo T (2010). Structural determinants of PERK inhibitor potency and selectivity. Chem Biol Drug Des.

[72] Harding HP, Zyryanova AF, Ron D (2012). Uncoupling proteostasis and development in vitro with a small molecule inhibitor of the pancreatic endoplasmic reticulum kinase, PERK. J Biol Chem.

[73] Atkins C, Liu Q, Minthorn E, Zhang SY, Figueroa DJ, Moss K, Stanley TB, Sanders B, Goetz A, Gaul N, Choudhry AE, Alsaid H, Jucker BM, Axten JM, Kumar R (2013). Characterization of a novel PERK kinase inhibitor with antitumor and antiangiogenic activity. Cancer Res.

[74] Ye Y, Meyer HH, Rapoport TA (2001). The AAA ATPase Cdc48/p97 and its partners transport proteins from the ER into the cytosol. Nature.

[75] Dai RM, Chen E, Longo DL, Gorbea CM, Li CC (1998). Involvement of valosin-containing protein, an ATPase Co-purified with IkappaBalpha and 26 S proteasome, in ubiquitin-proteasome-mediated degradation of IkappaBalpha. J Biol Chem.

[76] Li JM, Wu H, Zhang W, Blackburn MR, Jin J (2014). The p97-UFD1L-NPL4 protein complex mediates cytokine-induced IkappaBalpha proteolysis. Mol Cell Biol.

[77] Yamamoto S, Tomita Y, Nakamori S, Hoshida Y, Iizuka N, Okami J, Nagano H, Dono K, Umeshita K, Sakon M, Ishikawa O, Ohigashi H, Aozasa K, Monden M (2004). Valosin-containing protein (p97) and Ki-67 expression is a useful marker in detecting malignant behavior of pancreatic endocrine neoplasms. Oncology.

[78] Yamamoto S, Tomita Y, Hoshida Y, Iizuka N, Monden M, Yamamoto S, Iuchi K, Aozasa K (2004). Expression level of valosin-containing protein (p97) is correlated with progression and prognosis of non-small-cell lung carcinoma. Ann Surg Oncol.

[79] Yamamoto S, Tomita Y, Hoshida Y, Nagano H, Dono K, Umeshita K, Sakon M, Ishikawa O, Ohigashi H, Nakamori S, Monden M, Aozasa K (2004). Increased expression of valosin-containing protein (p97) is associated with lymph node metastasis and prognosis of pancreatic ductal adenocarcinoma. Ann Surg Oncol.

[80] Yamamoto S, Tomita Y, Hoshida Y, Takiguchi S, Fujiwara Y, Yasuda T, Yano M, Nakamori S, Sakon M, Monden M, Aozasa K (2003). Expression level of valosin-containing protein is strongly associated with progression and prognosis of gastric carcinoma. J Clin Oncol.

[81] Yamamoto S, Tomita Y, Nakamori S, Hoshida Y, Nagano H, Dono K, Umeshita K, Sakon M, Monden M, Aozasa K (2003). Elevated expression of valosin-containing protein (p97) in hepatocellular carcinoma is correlated with increased incidence of tumor recurrence. J Clin Oncol.

[82] Yamamoto S, Tomita Y, Uruno T, Hoshida Y, Qiu Y, Iizuka N, Nakamichi I, Miyauchi A, Aozasa K (2004). Expression level of valosin-containing protein (p97) is associated with prognosis of esophageal carcinoma. Clin Cancer Res.

[83] Yamamoto S, Tomita Y, Uruno T, Hoshida Y, Qiu Y, Iizuka N, Nakamichi I, Miyauchi A, Aozasa K (2005). Increased expression of valosin-containing protein (p97) is correlated with disease recurrence in follicular thyroid cancer. Ann Surg Oncol.

[84] Fessart D, Marza E, Taouji S, Delom F, Chevet E (2013). P97/CDC-48: proteostasis control in tumor cell biology. Cancer Lett.

[85] Verma R, McDonald H, Yates JR, Deshaies RJ (2001). Selective degradation of ubiquitinated Sic1 by purified 26S proteasome yields active S phase cyclin-Cdk. Mol Cell.

[86] Franz A, Ackermann L, Hoppe T (1843). Create and preserve: proteostasis in development and aging is governed by Cdc48/p97/VCP. Biochim Biophys Acta.

[87] Meyer H, Bug M, Bremer S (2012). Emerging functions of the VCP/p97 AAA-ATPase in the ubiquitin system. Nat Cell Biol.

[88] Carvalho P, Goder V, Rapoport TA (2006). Distinct ubiquitin-ligase complexes define convergent pathways for the degradation of ER proteins. Cell.

[89] Verma R, Oania R, Fang R, Smith GT, Deshaies RJ (2011). Cdc48/p97 mediates UV-dependent turnover of RNA Pol II. Mol Cell.

[90] Beskow A, Grimberg KB, Bott LC, Salomons FA, Dantuma NP, Young P (2009). A conserved unfoldase activity for the p97 AAA-ATPase in proteasomal degradation. J Mol Biol.

[91] Verma R, Oania RS, Kolawa NJ, Deshaies RJ (2013). Cdc48/p97 promotes degradation of aberrant nascent polypeptides bound to the ribosome. eLife.

[92] Brandman O, Stewart-Ornstein J, Wong D, Larson A, Williams CC, Li GW, Zhou S, King D, Shen PS, Weibezahn J, Dunn JG, Rouskin S, Inada T, Frost A, Weissman JS (2012). A ribosome-bound quality control complex triggers degradation of nascent peptides and signals translation stress. Cell.

[93] Defenouillère Q, Yao Y, Mouaikel J, Namane A, Galopier A, Decourty L, Doyen A, Malabat C, Saveanu C, Jacquier A, Fromont-Racine M (2013). Cdc48-associated complex bound to 60S particles is required for the clearance of aberrant translation products. Proc Natl Acad Sci U S A.

[94] Buchan JR, Kolaitis RM, Taylor JP, Parker R (2013). Eukaryotic stress granules are cleared by autophagy and Cdc48/VCP function. Cell.

[95] Ju JS, Fuentealba RA, Miller SE, Jackson E, Piwnica-Worms D, Baloh RH, Weihl CC (2009). Valosin-containing protein (VCP) is required for autophagy and is disrupted in VCP disease. J Cell Biol.

[96] Ju JS, Miller SE, Hanson PI, Weihl CC (2008). Impaired protein aggregate handling and clearance underlie the pathogenesis of p97/VCP-associated disease. J Biol Chem.

[97] Tresse E, Salomons FA, Vesa J, Bott LC, Kimonis V, Yao TP, Dantuma NP, Taylor JP (2010). VCP/p97 is essential for maturation of ubiquitin-containing autophagosomes and this function is impaired by mutations that cause IBMPFD. Autophagy.

[98] Pandey UB, Nie Z, Batlevi Y, McCray BA, Ritson GP, Nedelsky NB, Schwartz SL, DiProspero NA, Knight MA, Schuldiner O, Padmanabhan R, Hild M, Berry DL, Garza D, Hubbert CC, Yao TP, Baehrecke EH, Taylor JP (2007). HDAC6 rescues neurodegeneration and provides an essential link between autophagy and the UPS. Nature.

[99] Magnaghi P, D'Alessio R, Valsasina B, Avanzi N, Rizzi S, Asa D, Gasparri F, Cozzi L, Cucchi U, Orrenius C, Polucci P, Ballinari D, Perrera C, Leone A, Cervi G, Casale E, Xiao Y, Wong C, Anderson DJ, Galvani A, Donati D, O'Brien T, Jackson PK, Isacchi A (2013). Covalent and allosteric inhibitors of the ATPase VCP/p97 induce cancer cell death. Nat Chem Biol.

[100] Chou TF, Brown SJ, Minond D, Nordin BE, Li K, Jones AC, Chase P, Porubsky PR, Stoltz BM, Schoenen FJ, Patricelli MP, Hodder P, Rosen H, Deshaies RJ (2011). A reversible inhibitor of the AAA ATPase p97, DBeQ, impairs both ubiquitin-dependent and autophagic protein clearance pathways. Proc Natl Acad Sci U S A.

[101] Acharya P, Liao M, Engel JC, Correia MA (2011). Liver cytochrome P450 3A endoplasmic reticulum-associated degradation: a major role for the p97 AAA ATPase in cytochrome p450 3A extraction into the cytosol. J Biol Chem.

[102] Piccirillo R, Goldberg AL (2012). The p97/VCP ATPase is critical in muscle atrophy and the accelerated degradation of muscle proteins. EMBO J.

[103] Chou TF, Li K, Frankowski KJ, Schoenen FJ, Deshaies RJ (2013). Structure-activity relationship study reveals ML240 and ML241 as potent and selective inhibitors of p97 ATPase. Chem Med Chem.

[104] Wang Q, Li L, Ye Y (2008). Inhibition of p97-dependent protein degradation by Eeyarestatin I. J Biol Chem.

[105] Bursavich MG, Parker DP, Willardsen JA, Gao ZH, Davis T, Ostanin K, Robinson R, Peterson A, Cimbora DM, Zhu JF, Richards B (2010). 2-Anilino-4-aryl-1,3-thiazole inhibitors of valosin-containing protein (VCP or p97). Bioorg Med Chem Lett.

[106] Brown SJ, Chou TF, Deshaies R, Roberts E, Guerrero M, Minond D, Mercer BA, Hodder P, Rosen HR (2010). Probe report for P97/cdc48 inhibitors. Probe Reports from the NIH Molecular Libraries Program.

[107] Sasazawa Y, Kanagaki S, Tashiro E, Nogawa T, Muroi M, Kondoh Y, Osada H, Imoto M (2012). Xanthohumol impairs autophagosome maturation through direct inhibition of valosin-containing protein. ACS Chem Biol.

[108] Yi P, Higa A, Taouji S, Bexiga MG, Marza E, Arma D, Castain C, Le Bail B, Simpson JC, Rosenbaum J, Balabaud C, Bioulac-Sage P, Blanc JF, Chevet E (2012). Sorafenib-mediated targeting of the AAA(+) ATPase p97/VCP leads to disruption of the secretory pathway, endoplasmic reticulum stress, and hepatocellular cancer cell death. Mol Cancer Ther.

[109] Ikeda HO, Sasaoka N, Koike M, Nakano N, Muraoka Y, Toda Y, Fuchigami T, Shudo T, Iwata A, Hori S, Yoshimura N, Kakizuka A (2014). Novel VCP modulators mitigate major pathologies of rd10, a mouse model of retinitis pigmentosa. Sci Rep.

[110] Kang MJ, Wu T, Wijeratne EM, Lau EC, Mason DJ, Mesa C, Tillotson J, Zhang DD, Gunatilaka AA, La Clair JJ, Chapman E (2014). Functional chromatography reveals three natural products that target the same protein with distinct mechanisms of action. Chembiochem.

[111] Chou TF, Deshaies RJ (2011). Quantitative cell-based protein degradation assays to identify and classify drugs that target the ubiquitin-proteasome system. J Biol Chem.

[112] Johnson ES, Ma PC, Ota IM, Varshavsky A (1995). A proteolytic pathway that recognizes ubiquitin as a degradation signal. J Biol Chem.

[113] Dantuma NP, Lindsten K, Glas R, Jellne M, Masucci MG (2000). Short-lived green fluorescent proteins for quantifying ubiquitin/proteasome-dependent proteolysis in living cells. Nat Biotechnol.

[114] Kimbrel EA, Davis TN, Bradner JE, Kung AL (2009). In vivo pharmacodynamic imaging of proteasome inhibition. Mol Imaging.

[115] Polucci P, Magnaghi P, Angiolini M, Asa D, Avanzi N, Badari A, Bertrand J, Casale E, Cauteruccio S, Cirla A, Cozzi L, Galvani A, Jackson PK, Liu Y, Magnuson S, Malgesini B, Nuvoloni S, Orrenius C, Sirtori FR, Riceputi L, Rizzi S, Trucchi B, O'Brien T, Isacchi A, Donati D, D'Alessio R (2013). Alkylsulfanyl-1,2,4-triazoles, a new class of allosteric valosine containing protein inhibitors: synthesis and structure-activity relationships. J Med Chem.

[116] Radhakrishnan SK, Lee CS, Young P, Beskow A, Chan JY, Deshaies RJ (2010). Transcription factor Nrf1 mediates the proteasome recovery pathway after proteasome inhibition in mammalian cells. Mol Cell.

[117] Steffen J, Seeger M, Koch A, Kruger E (2010). Proteasomal degradation is transcriptionally controlled by TCF11 via an ERAD-dependent feedback loop. Mol Cell.

[118] Radhakrishnan SK, den Besten W, Deshaies RJ (2014). p97-dependent retrotranslocation and proteolytic processing govern formation of active Nrf1 upon proteasome inhibition. eLife.

[119] Zhang Y, Ren Y, Li S, Hayes JD (2014). Transcription factor Nrf1 is topologically repartitioned across membranes to enable target gene transactivation through its acidic glucose-responsive domains. PLoS One.

[120] Sha Z, Goldberg AL (2014). Proteasome-mediated processing of Nrf1 is essential for coordinate induction of all proteasome subunits and p97. Curr Biol.

[121] Wang W, Chan JY (2006). Nrf1 is targeted to the endoplasmic reticulum membrane by an N-terminal transmembrane domain. Inhibition of nuclear translocation and transacting function. J Biol Chem.

[122] Auner HW, Moody AM, Ward TH, Kraus M, Milan E, May P, Chaidos A, Driessen C, Cenci S, Dazzi F, Rahemtulla A, Apperley JF, Karadimitris A, Dillon N (2013). Combined inhibition of p97 and the proteasome causes lethal disruption of the secretory apparatus in multiple myeloma cells. PLoS One.

[123] **ClinicalTrials.gov, search terms "CB-5083" and "cleave".** In [clinicaltrials.gov/ct2/results?term = cb-5083 + AND + cleave&Search = Search]

